# Comparison of E-Cadherin Expression in Oral Verrucous Carcinoma and Normal Oral Mucosa: An Immunohistochemical Study

**DOI:** 10.7759/cureus.74634

**Published:** 2024-11-27

**Authors:** Pratijya Raj, Manish Bhargava, Anchal Varshney

**Affiliations:** 1 Department of Oral Pathology and Microbiology, Manav Rachna Dental College, Faridabad, IND

**Keywords:** cell to cell adhesion molecule, e-cadherin, immunohistochemistry(ihc), tumour, verrucous carcinoma

## Abstract

Introduction

Oral verrucous carcinoma (OVC), a low-grade variation of oral squamous cell carcinoma (OSCC), is distinguished by endophytic development and a pebbly, mammillated surface. OVC, often referred to as Ackerman's tumor, has been known to involve lymph nodes but rarely spreads to regional and distant locations; when the primary tumor grows, it frequently involves surrounding tissues. Histopathologically, it has a thicker basement membrane, many reduplications, and a large area of inflammatory infiltration that resembles OSCC. Therefore, precise histological diagnosis of verrucous carcinoma is crucial as helps in identifying tumors with a higher propensity to develop into OSCC. In cancer progression, the epithelial-mesenchymal transition (EMT) is the most important stage. One factor influencing EMT is epithelial cadherin (E-cadherin). Thus, it is imperative to identify indicators that can facilitate the detection of lesions with the potential to progress into OSCC.

Aim

This study aimed to identify the expression of E-cadherin in normal mucosa and verrucous carcinoma and determine its role in the progression of the lesion.

Methodology

A total of 15 subjects with normal mucosa and 15 subjects with verrucous carcinoma were histopathologically examined and confirmed. Tissue sections were stained immunohistochemically with E-cadherin, utilizing the normal mucosa as the control group.

Results

When comparing normal oral mucosa to oral verrucous carcinoma, a decrease in E-cadherin expression was noted. Nonetheless, a statistically significant connection was identified between clinical parameters and E-cadherin expression solely concerning gender.

Conclusion

Through this study, we have attempted to assess and correlate the expression of E-cadherin between OVC and normal oral mucosa with clinical parameters. Furthermore, compared to normal oral mucosa, there was a significant decrease in the expression of E-cadherin in OVC. While further research with an extensive panel of biomarkers and a larger sample size could yield a greater understanding of carcinogenesis mechanisms, E-cadherin serves as a significant marker in partially evaluating the carcinogenic process of oral cancer.

## Introduction

Oral verrucous carcinoma (OVC), a low-grade variety of oral squamous cell carcinoma (OSCC), is characterized by its distinctive endophytic development and pebbled, mamillated surface. OVC represents 2-16% of all oral cancers. Commonly referred to as Ackerman’s tumor, OVC has been documented to involve lymph nodes and rarely metastasize to remote locations, with neighboring structures becoming implicated as the main tumor enlarges over time. Histopathologically, OVC displays a thicker basement membrane with reduplication in many locations and significant inflammatory infiltrate, similar to OSCC [1]. Consequently, an accurate histological diagnosis of verrucous carcinoma is essential, as it facilitates the recognition of tumors with an increased likelihood of advancing to OSCC [2].

Epithelial-mesenchymal transition (EMT) represents a multi-step process that enables epithelial cells to undergo a series of biochemical changes, resulting in their transformation into a mesenchymal phenotype. These epithelial cell-to-cell interactions are crucial for maintaining cellular polarity and tissue integrity. The junctional complex, consisting of tight junctions, adherens junctions, and desmosomes, plays a key role in this process. Cadherins, the primary adhesion molecules in adherens junctions, are essential for regulating the overall activity of the junctional complex. Modifications in the interaction between the basal cell layers and the basement membrane, such as enhanced migratory capacity, increased invasiveness, heightened resistance to apoptosis, and significantly elevated production of extracellular matrix components, are hallmarks of cancer progression. Most cancers, including OSCC, undergo invasion and metastasis, which requires the loss of cell-to-cell adhesion [3]. Therefore, identifying a reliable marker that can help detect lesions with the potential to progress into OSCC is critical for early diagnosis and effective treatment.

E-cadherin, a 120-kDa calcium-dependent cell-surface glycoprotein, plays a crucial role in mediating adhesion between epithelial cells, thereby inhibiting cell proliferation. It functions as a key biomarker for suppressing growth and proliferation, contributing significantly to the maintenance of tissue architecture and cellular homeostasis. The suppression of E-cadherin expression is regarded as one of the main molecular events responsible for dysfunction in cell-cell adhesion. The expression of E-cadherin has been quite variable in various studies on oral and extraoral lesions [4]. 

Thus, the objective of this work was to examine the expression of E-cadherin and assess its functional role in OVC and normal oral mucosa, to enhance the understanding of its importance in tumor growth and cellular differentiation.

## Materials and methods

This was a study conducted at Sri Dharmasthala Manjunatheswara College of Dental Sciences & Hospital, Dharwad, Karnataka, India, from February 2018 to January 2019. The study was approved by Sri Dharmasthala Manjunatheswara College of Dental Sciences & Hospital, Dharwad, Karnataka, India (approval number: 2016/P/OP/50).

The study included 30 histopathologically verified cases, comprising 15 cases of normal oral mucosa (Group 1) and 15 cases of oral verrucous carcinoma (Group 2). Participants were of all age groups and of both genders. Individuals without a history of habit or who were not histologically verified were excluded.

The minimum sample size of 15 OVC and 15 normal mucosa was found to be sufficient to obtain alpha of 0.05, a power of 80%, and effect size of 0.2 (Cohen’s d).

Two sections were extracted from the paraffin-embedded tissue blocks of primary lesions in Group 2. One part was stained with hematoxylin and eosin for general histopathological evaluation while the other section underwent immunohistochemistry to confirm the histopathological diagnosis of the cases. The paraffin-embedded tissue blocks for the investigation were obtained from the archives of the Department of Oral and Maxillofacial Pathology and Microbiology at Sri Dharmasthala Manjunatheswara College of Dental Sciences & Hospital. Normal oral mucosal tissues for Group 1 were procured after the surgical extraction of impacted teeth, with the full consent of the patients. Clinical information of patients was retrieved from the department's database. The exclusion criteria encompassed tissue samples with inadequate epithelial or connective tissue depth, folded or necrotic regions, recurrent instances of oral verrucous carcinoma, and cases devoid of acceptable clinical data.

Procedure

Immunohistochemical Staining

For immunohistochemical staining, the primary antibody used was E-cadherin rabbit monoclonal antibody (Clone EPR4120, IgG immunoglobulin). Staining was conducted in accordance with the manufacturer's instructions (BioGenex Life Sciences Pvt Ltd, Adibatla, Telangana, India).

Staining interpretation:As observed in our positive control sections, the presence of a brown-colored end product at the target antigen location was regarded as positive immunoreactivity. Membranous staining, membranous, and cytoplasmic staining for E-cadherin were considered positive. Cytoplasmic staining alone and unstained cells for E-cadherin were considered negative.

Defining the field for cell counting:In each group, areas of the primary lesions were scanned using low-power magnification. At a greater magnification (x40), folded sections and areas that were not recognizable for counting were noted and excluded from counting.

Cell counting: Counting was done using a binocular light microscope with high-power magnification (x40). Immunoreactivity was evaluated quantitatively on the basis of distribution and staining intensity. In normal oral mucosa and oral verrucous carcinoma, the total number of cells counted was 1000 cells. The percentage of positive tumor cells was obtained by the simple formula shown below.

Total number of positive tumour cells:

M% = $\frac{M}{1000}\times 100$

(M + C): M% = $\frac{M+C}{1000}\times 100$

where M= Membrane, (M + C) = Mixed i.e. membrane and cytoplasm

The total number of tumor-positive cells was categorized based on the Intensity Reactive Score (IRS) [5] as follows: (i) 0% = 0; (ii) 1-30% = 1; (iii) 30-60% = 2; (iv) >60% = 3.

Statistical analysis

All samples were independently assessed by two observers to reduce observer bias in quantifying positive cells. Due to the absence of a statistically significant difference between the values recorded by the two observers for positive staining, the results from the first observer were chosen for further investigation (Table 1).

**Table 1:** Observer values for E-cadherin expression in the cases

Observer 1	Observer 2	P-value
Group 1: 97.26%	Group 1: 97.6%	0.53

The data were evaluated utilizing the Chi-square test, with a p-value of less than 0.05 being considered statistically significant. The data were analyzed by IBM SPSS Statistics for Windows, Version 20.0 (Released 2011; IBM Corp., Armonk, New York, United States).

## Results

The characteristics of the groups are given in Table 2. We examined and correlated the expression of E-cadherin in the two groups. The expression of E-cadherin was analyzed in connection with clinical characteristics, including gender, age, location, and behaviors. Our data revealed that, except for gender, there were no statistically significant relationships between E-cadherin expression and the other clinical indicators (Table 2).

**Table 2:** Characteristics of the two groups *Chi-square value is not applicable as parameters are 0

Characteristics	Group 1	Group 2	Chi-square value	p-value
Gender			3.472	0.002
Male	6 (40%)	12 (80%)		
Female	9 (60%)	3 (20%)		
Age (years)			NA*	NA
>50	15 (100%)	15 (100%)		
= <50	0 (0%)	0 (0%)		
Site				
Buccal Mucosa	15 (100%)	9 (60%)	NA*	NA
Lip	0 (0%)	2 (13.3%)		
Mixed	0 (0%)	4 (26.6%)		
Risk-related Habits			NA*	NA
No habits	15 (100%)	3 (20%)		
Tobacco chewing	0 (0%)	10 (66.7%)		
Smoking	0 (0%)	2 (13.3%)		

Immunohistochemistry

**Figure 1:**
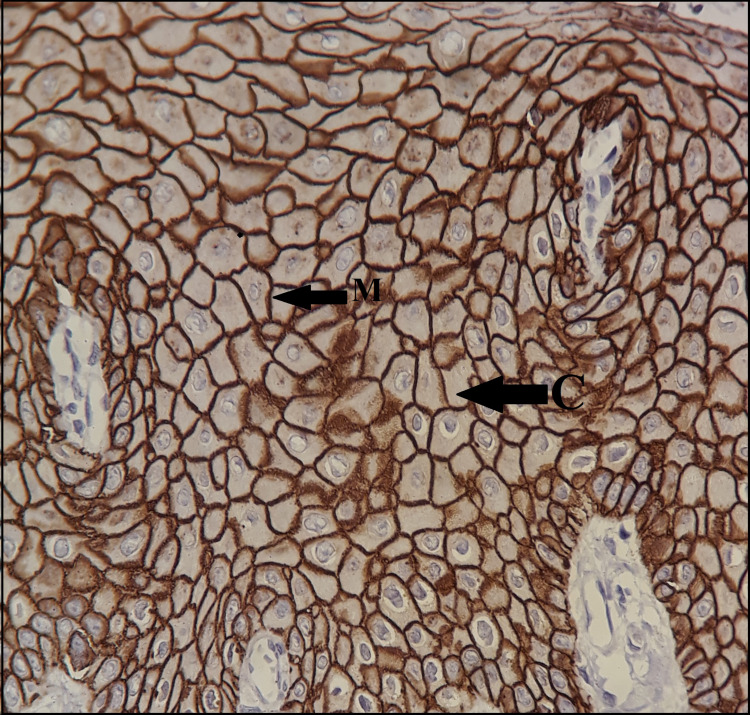
Membranous (M) and cytoplasmic (C) immunostaining in basal and superficial layer of normal oral epithelium (Magnification: x40, IHC: E-cadherin). IHC: immunohistochemistry

The presence of a brown-hued end product at the target antigen location showed positive staining. The expression of E-cadherin was assessed according to its localization pattern, which encompassed membranous, cytoplasmic, mixed (both membranous and cytoplasmic), and the presence or absence of staining (Figure 1).

**Figure 2:**
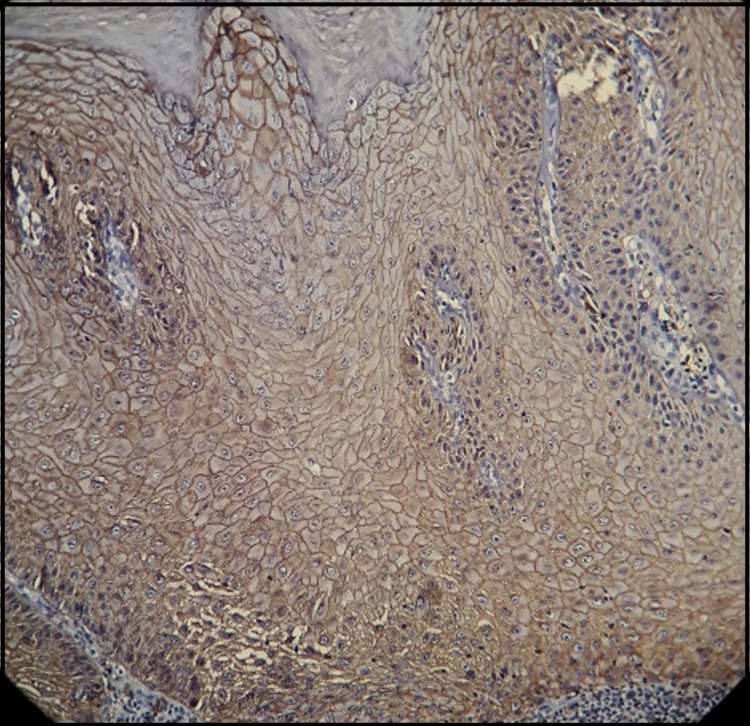
Verrucous carcinoma showing stratified squamous epithelium with pushing broad rete ridge showing loss of expression of E-cadherin in basal cell layer (Magnification: x10 inset, IHC: E-cadherin). IHC: immunohistochemistry

The expression of E-cadherin was subsequently compared between the two study groups. A substantial reduction in E-cadherin expression was found from normal mucosa to verrucous carcinoma (Figure 2).

When statistically evaluated, no significant difference was noted in E-cadherin expression between normal mucosa and verrucous carcinoma. Table 3 shows the overall comparison between normal mucosa and verrucous carcinoma for the expression of E cadherin.

**Table 3:** Overall comparison between the groups for the expression of E cadherin

Dependent Variable	Groups	no. of cases in each group	Mean	p value
% of positive staining	1	15	97.26	0.071
	2	15	90.51	

## Discussion

Cellular adhesion is essential in both healthy and pathological states, supporting the appropriate functioning of epithelial tissues via interactions with the extracellular matrix and intercellular connections [6]. Cell adhesion molecules, including cadherins, integrins, selectins, and members of the immunoglobulin superfamily, govern the epithelial phenotype by promoting homophilic cell attachment and preserving cellular polarity [7].

Cadherins constitute a substantial family of molecules that facilitate cell-to-cell adhesion via calcium-dependent homophilic interactions. E-cadherin, a traditional cadherin, is a crucial element of adherens junctions, functioning as a 120 kD glycoprotein that promotes epithelial cell adhesion through homotypic, calcium-dependent interactions [8]. This molecule is crucial for preserving epithelial integrity, and facilitating tissue morphogenesis, differentiation, and cell segregation, all of which are vital for the form and function of epithelial tissues [9].

Consistent strong pericellular staining of E-cadherin has been detected in the basal, suprabasal, and prickle cell layers of normal and hyperplastic epithelium, as shown in numerous studies [10]. Conversely, the keratinizing superficial layers have a deficiency in E-cadherin expression [10]. The decrease in E-cadherin expression is believed to contribute to the proper desquamation of epithelial cells, although the precise mechanisms governing its expression in the top layers are not well understood. Tyrosine phosphorylation during normal cell migration might trigger the disassembly of the E-cadherin-catenin complex, resulting in the delocalization of membrane-associated E-cadherin from adherens junctions. The phosphorylation of the E-cadherin-catenin complex, especially β-catenin, primarily mediates this process [11].

OVC, a subtype of squamous cell carcinoma, is typically less malignant than other variants of this disease and represents 2-16% of all oral carcinoma. Clinically, the lesion typically exhibits a pebbly, mamillated, or rugal-folded morphology, characterized by deep cleft-like regions that delineate these folds [1]. Microscopically, it is marked by a significant inflammatory response in the adjacent tissues, notable surface keratinization, and the downward extension of club-shaped projections of hyperplastic epithelium. These epithelial projections extend into the adjacent tissues without deeply penetrating them, frequently preserving an unbroken basement membrane. In mature stages, OVC typically invades neighboring structures with low mitotic activity, exhibits slow growth, and infrequently metastasizes [1].

The rapid growth of tumors and the initiation of metastasis in several carcinoma types, including those of the head and neck, have been associated with aberrant E-cadherin expression [12]. E-cadherin is crucial for cell adhesion, and its impairment is frequently linked to heightened tumor invasiveness and metastatic capability.

The present study found membrane, cytoplasmic, and solely cytoplasmic immunostaining of E-cadherin, consistent with the results of Sridevi and associates [12]. Multiple variables may influence the modified localization of E-cadherin, including enhanced production rate, failure to translocate, or inability to bind to the cell membrane. This positional change may correlate with staining patterns observed in non-cancerous situations, wherein the desmosomal glycoprotein in membrane vesicles experiences endocytosis and remains aggregated with plaque components due to the disintegration of intercellular connections. As these membrane vesicles deteriorate, the cytoplasmic staining gets dispersed.

The current study revealed that the majority of participants in both categories were men, aged over 50. This was perhaps due to the greater prevalence of habitual risk factors such as tobacco and alcohol consumption among men in India compared to women [13,14]. Kruse et al. observed that few studies have indicated a majority of female participants [15]. This may result from a change in behavioral patterns, as an increasing proportion of young women are being exposed to alcohol and tobacco use.

The buccal mucosa was determined to be the predominant site for the majority of lesions, with supplementary involvement observed in the lip, gingiva, and floor of the mouth. The research indicates that the preference for buccal mucosa may stem from extended exposure to tobacco and its components, which serve as a continual source of mucosal irritation and lesion development. Comparable results were documented by Kristofelc et al. [16]. This investigation found no significant association between the lesion site and E-cadherin expression, aligning with the results published by Andrews et al. [17]. Conversely, studies conducted by Tanaka et al. identified the tongue as the principal site of lesions, which differs from the current study, and similarly found no link between lesion location and E-cadherin expression [18]. This underscores the heterogeneity in lesion locations among various studies; however, the absence of association with E-cadherin expression remains constant.

According to Tang et al., E-cadherin expression in OVC (83.3%) was markedly lower than in poorly differentiated squamous cell carcinoma (37.5%) [19]. Klieb et al. reported widespread membranous staining across the epithelium in all instances of OVC and oral verrucous hyperplasia, indicating that modifications in adhesion molecules may be indicative of advanced stages of carcinogenesis [20]. These findings underscore the significance of E-cadherin alteration in tumor advancement.

Our study revealed no significant difference in E-cadherin expression between normal mucosa and verrucous carcinoma. This study indicates that, despite the unique histological characteristics of verrucous carcinoma, the expression of E-cadherin may not significantly differ from that of normal oral mucosa. This outcome may suggest that E-cadherin's efficacy as a biomarker in differentiating between these two situations is restricted, necessitating additional exploration of alternative markers or factors affecting tumor behavior.

The E-cadherin protein, produced by the *CDH1* gene, may be inactivated in human malignant tumors via various processes, including somatic mutations, genetic abnormalities, activation of transcriptional repressors, abnormal protein processing, and promoter hypermethylation. The reduction or lack of E-cadherin expression promotes EMT, a process in which epithelial cells gain motility and invasiveness, hence increasing their capacity to invade neighboring tissues and contribute to tumor invasion and metastasis [21]. E-cadherin expression is widely acknowledged as a vital marker for EMT in tumor growth, highlighting its importance in cancer biology and its potential as a therapeutic target.

Zhang et al. showed that the downregulation of E-cadherin expression adversely affects cell adhesion, resulting in heightened infiltration, dispersion, and metastasis of tumor cells [22]. This discovery indicates that E-cadherin is essential for preserving epithelial integrity and inhibiting malignant development. Thus, E-cadherin expression may function as a significant prognostic marker for individuals with verrucous carcinoma. Further research is necessary to confirm these findings and determine the clinical significance of E-cadherin as a prognostic factor in this situation.

Limitations of the study

Several limitations must be considered in our study. Firstly, due to the small sample size, the generalizability of the findings could be affected. Using a panel of markers instead of only one IHC marker could improve the study's outcomes. Additionally, variations in tissue processing and IHC staining techniques could introduce potential sources of error.

## Conclusions

OVC is a low-grade variant of squamous cell carcinoma, but it may transform into frank OSCC. Through this study, we have attempted to assess and correlate the expression of E-cadherin between OVC and normal oral mucosa. Reduced expression in OVC suggests a potential disruption in epithelial integrity, facilitating tumor invasion and progression into OSCC.

Our study emphasizes the necessity for additional investigation in this compelling domain. Subsequent research may augment the comprehension of the molecular mechanisms underlying tumor dissemination, facilitating the formulation of more efficacious therapeutic methods to enhance patient survival. Moreover, extensive research employing various biomarker panels and sophisticated approaches may yield an enhanced understanding of the dynamics of OVC.
